# Reciprocal Association Between Cognitive Function and Oral Health: A Systematic Review and Meta-Analysis

**DOI:** 10.1093/geroni/igaa057.514

**Published:** 2020-12-16

**Authors:** Xiang Qi, Zheng Zhu, Bei Wu

**Affiliations:** 1 New York University, jersey city, New Jersey, United States; 2 Fudan University, Shanghai, China; 3 New York University, New York, New York, United States

## Abstract

Increasing evidence suggests that there is a linkage between cognitive function and oral health. However, there are few systematic reviews with meta-analysis have been conducted to evaluate the strength of this association. Moreover, existing studies usually focused on unidirectional associations between cognitive function and oral health; no research has demonstrated this inter-relationship in a longitudinal study. This study aims to systematically assess the magnitude of the bidirectional association between oral health and cognitive decline for studies using longitudinal data. Six international databases were searched up until December 31, 2019. Random-effects pooled Risk Ratios (RRs) with 95% confidence intervals (CIs) were calculated. The grading of recommendations assessment, development, and evaluation (GRADE) system was used to assess the quality of evidence. From 13,251 potentially eligible articles, 54 longitudinal studies were included in the systematic review and 18 were in meta-analysis. Meta-analysis was performed for tooth loss and periodontitis disease. Random effects analysis showed, with statistically low heterogeneity, Risks of cognitive decline included suboptimal dentition (<20 teeth) (RR 1.44, 95% CI 1.03-3.65) and periodontitis diseases (RR 1.48, 95% CI 1.22-1.81). Cognitive decline was a risk factor for tooth loss (RR 1.54, 95% CI 1.23-9.69). The overall quality of evidence, however, was rated as very low. The result of this review highlights that cognitive decline is a risk factor for poor oral health, and older adults with suboptimal oral health appear to have an increased risk of cognitive impairment. More studies with rigorous designs are needed to further examine this association.

